# Retraction of invasive *Spartina alterniflora* and its effect on the habitat loss of endangered migratory bird species and their decline in YNNR using remote sensing technology

**DOI:** 10.1002/ece3.6971

**Published:** 2020-11-09

**Authors:** Onyedikachi Kingsley Okoye, Huan Li, Zheng Gong

**Affiliations:** ^1^ College of Harbor, Coastal and Offshore Engineering Hohai University Nanjing China

**Keywords:** endangered birds, habitat loss, remote sensing, *Spartina alterniflora*

## Abstract

Wetland environment and habitat loss increase the rate of biodiversity decline and affect our ecosystems. Yancheng National Nature Reserve (YNNR) is a protected area dedicated to endangered migratory bird species to overwinter. However, it currently has a record low influx of migrating birds and might therefore be losing its founding purpose. We used remote sensing technology to assess and quantify the impacts and effects of invasive halophytes *Spartina alterniflora* in the habitat loss and shrinkage of endangered bird wintering habitat from 2003 to 2018. We also attempted to ascertain the causes and triggers of avian population decline and its relationship with habitat loss, as these phenomena threaten and endanger species both locally and globally. Our study shows how YNNR has lost about 80% of migratory bird habitat to invasive *S. alterniflora* and *Phragmites australis*, a native halophyte plant in the reserve. Furthermore, shoreline erosion triggered the retraction of *S. alterniflora* and its backward growth toward *Suaeda Salsa*, the preferred foraging habitat for migratory birds in the zone, which is a possible cause of their decline.

## Graphical abstract

Remote sensing, habitat loss, and endangered bird.

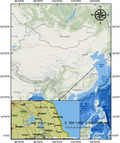

## 1 INTRODUCTION

Wetlands are the primary role player in the sustenance of our fragile and delicate ecosystems, marine habitats, estuaries, and ground and surface water resources. However, wetlands are quite sensitive to loss, degradation, and erosion, depending on the activities and exploitation done on it. Wetland types include marshes, peat, fen, and water bodies like swamp, saltwater, freshwater, and brackish water. Varieties of plants and animal species live, breed, and forage in the wetlands.

*Spartina alterniflora* is simply an invasive perennial rhizomatous deep‐rooted salt marsh grass, which plays an essential role in ecological function in its native ecosystems (Xiao et al., 2010). *Spartina alterniflora* is native to the eastern coast of North America. Invasive plants are plants that are deliberately or inadvertently introduced into any habitat where they have not evolved. Invasive species can spread into various habitat and beyond their native range (Zheng et al., 2016). They are believed to cause havoc to the environment and human welfare after being introduced (Ehrenfeld, 2010). Invasive species have increased and grown repeatedly during the past decades and show no saturation (Seebens et al., 2017). These are due to anthropogenic effects such as agriculture, international travel, and trade (transoceanic ships transporting and carrying an immeasurable number of organisms on their hulls, bow, bow thrusters, keels, thrusters, and ballast tanks) (Lin et al., 2011; Westphal et al., 2008). However, invasive species conventionally have no limitation or natural enemies to limit their growth and expansion, as they can spread over long distances when introduced to a new environment (Ricciardi, 2013). *Spartina alterniflora* cordgrass has spread swiftly to estuaries and coastal marshes all around the world (Zheng, 2016). *Spartina alterniflora* was deliberately and intentionally introduced to China coastlands in 1979 to help curb and reduce many wetland and coastal activities such as erosion control, soil amelioration, sediment stabilization, land reclamation, marsh restoration, and dike protection (Li et al., 2010). *Spartina alterniflora* plays an essential role in reducing soil salt content in coasts and could be attributed to its central role in controlling tidal flooding (Yang et al., 2019).

Yancheng National Natural Reserve (YNNR) is located at Yancheng Coastal Wetlands, Yancheng city, Jiangsu Province, China. YNNR is a devoted and vulnerable reserved biosphere and the largest coastal wetlands reserve in China (Zhu et al., 2004). Hydrochloric tolerant marshes dominate the vegetation in YNNR. YNNR provides an ecosystem for stopover shorebirds to feed on benthic invertebrates during their annual migration (Barter, 2012; He‐Bo, 2017). YNNR was mapped and established in 1984. First, YNNR was recognized as a provincial reserve. Second, the reserve was recognized and classified as a national nature reserve in 1992. Furthermore, it was welcome and included in the International Man and Biosphere Reserve Network of UNESCO in the same year. YNNR was consolidated in the Reserve Network of Northeast Asian Cranes in 1996. This region has the most significant number of shorebirds and threatened species population stopover (Studds et al., 2017). The principle aim of establishing this reserve is to provide a wintering ground for the protection and study of migratory endangered and threatened coastal rare bird species (Xu et al., 2014) such as red‐crowned cranes (*Grus japonensis*) (List, 2020). Nevertheless, red‐crowned cranes species are regarded as the world most vulnerable, threatened, endangered, and classified as a first‐grade‐protected migratory bird species (IUCN, 2014) by the International Union for Conversation of Nature's Red List of Threatened Species (IUCN Red List) whose sole mission is to report world's most comprehensive information on the status of animals, fungus, and plant species global extinction risk status. It has been reported about the troubling decline of shorebirds across the East Asian–Australasian Flyway (EAAF) (Bamford, 2008; Li et al., 2019).

Moreover, the formation, expansion, and invasion of *S. alterniflora* in YNNR have endangered the living space of other native saltmarshes and organisms (Wang, 2019). The vegetation in YNNR before the introduction of *S. alterniflora* was *Suaeda salsa*, Couch grass, and *Phragmites australis*. This vegetation spreads from the intertidal to supratidal surfaces. Currently, in YNNR, *S. alterniflora* has altered and changed the landscape pattern as it typically dominated the shorelines and most of the tidal flats in this reserve. YNNR shoreline has a severe erosion; the consistency of this shoreline erosion will change and affect the growth and behavior of *S. alterniflora*. If this problem continues to linger, *S. alterniflora* will continuously grow inwards (from the intertidal zone) and squeeze *S. salsa* saltmarsh habitat, and *P. australis* will do the same from inward (supratidal zone). Currently, the saltmarshes vegetation in YNNR is as follows from seashore to inland: *S. alterniflora*, *S. salsa*, and *P. australis*. *Spartina alterniflora* is currently displacing other native species, which provides a habitat for the breeding and foraging of migratory birds and endangered birds in the core zone of YNNR.

*Spartina alterniflora* is reported in this study to be expanding from the shores and growing with full force toward the inland of YNNR using remote sensing technology, which is quite unusual and strange since the introduction of *S. alterniflora* in YNNR and China coast. Research carried out in YNNR is mainly on the introduction, formation, growth, and expansion of *S. alterniflora* in the coast of Jiangsu where YNNR is located in Xiao et al. (2010) and Bamford (2008), the effects of *S. alterniflora* to wintering habitat of red‐crowned cranes in YNNR (Wang, 2019), on the classification of coastal vegetation in YNNR (Kang et al., 2013). Finally, in the mapping out reserves for the conversation of shorebirds and about the troubling decline of shorebirds across the East Asian–Australasian Flyway (EAAF) (Moores, 2016; Piersma, 2016), as this region has the most significant number of shorebird and threatened species population.

We considered and mapped out the habitat selections of red‐crowned cranes based on some literature reviews and reports of other researchers and statistics from Yancheng Reserve Administration Bureau and valuable historical records of past landscape conditions on habitat preference of red‐crowned cranes. Their habitat selection is related to food availability, disturbance agents, and sleeping places. Our literature reviewed used head counting by experienced water bird surveyors (Peng & Anderson, 2017). Also, statistical methods, binomial logistic regression models, remote sensing methods, the geographically weighted regression model (GWR), GPS, and telescopes in analyzing the spatial distribution of red‐crowned crane in their wintering habitat (Cao et al., 2015; Honghai & Yuewei, 2000; Kim et al., 2016; Liu, 2018; Ma et al., 1999; Wang et al., 2019). Most of these researches and surveys were carried during high tides, mainly when water birds were restricted to feeding or roosting (Bai et al., 2015; Choi, 2016).

Remote sensing technology is a technique used to record, observe, and store electromagnetic data, waves, or energies dissipated by a target object or from a given area (Van der Wiele et al., 2016). Remote sensed data from multiple sensors could maximize environmental monitoring. These sensors acquire imagery or data in varying conditions, multiple resolutions (such as spatial, spectral, and radiometric resolution) and multiple bandwidths (Melesse et al., 2007; Thenkabail et al., 2004). Besides, these sensors supply data readily in a wide range of scales such as pixel resolutions, bandwidths, radiometry, and band numbers (Tucker et al., 2005). Satellite and aerial photographs remote sensing come with numerous advantages such as revisiting a particular geographic area or place of interest on a regular cycle, which aids in facilitating data acquisition to reveal changing conditions over time (Randall, 2012). Thus, it allows understanding vegetation patterns and environmental changes of our terrestrial environment (Harvey & Hill, 2001), landscape conditions, and identifying the major causes of environmental degradation of coastal wetlands (Bustamante et al., 2016; Kirwan, 2013; Lee, 2006; Tangao et al., 2019). Remote sensing helps in substantial or regional‐scale research, hard to carry out by modeling or field observations (Venevsky, 2019). An example is a limitation in global sea‐rise consequences, in which there are no readily available models for its detection presently (Yang, 2013).

Studies carried out with remote sensing in Yancheng, Jiangsu, China, are mostly on the use of remote sensing technology to analyze, monitor, and explore the phenology of coastal vegetation at a substantial spatial and long time scale in the southern Yellow Sea (Jiangsu, Yancheng), China (Wu et al., 2018). Also, using remote sensing technology application to analyze coastal wetland degradation and its key restoration technologies in the coastal area of Jiangsu (Cui et al., 2018), Landsat Thematic Mapper (TM), (SPOT), and other multispectral sensors have been used in keeping track of invasive species (Laba, 2010) and in discovering the composition of wetland in landscapes that are heterogeneous (Wright, 2007). Landsat Thematic Mapper (TM)‐based classifications of forested wetland maps show higher accuracy of 82% in Maine, USA (Sader, 1995). Furthermore, it also shows a higher overall accuracy of (80%) when mapping riparian zones in xeric ecosystem of eastern Washington, and southeastern USA (Hewitt, 1990; Walsh, 2001), when compared to other passive and active spaceborne sensors (Bolstad, 1992; Corey Baker, 2006; Matthew Lebsock, 2014). However, the significant concerns in remote sensing are the huge time lapse on imagery acquisition and production of final wetland maps (Ramsey, 1997), thus the need for automated and reproducible wetland maps (Finlayson, 1995). An additional concern of remote sensing is in low accuracy classification of land covers and land use. These problems are attributed to mix pixels problems. Mixed pixels are often because most pixel resolution fails to correspond or tally with the spatial characteristics of the target (Mather, 1999). However, approaches like “soft”/fuzzy approach (Melesse et al., 2007; Wang, 1990) and linear spectral mixture analysis (LSMA) (Wu, 2005) of LU/LC classifications have been used in the mitigating problem of mixed pixels (Melesse et al., 2007).

The study objectives and focus are solely on

1. To ascertain the effects of *S. alterniflora* invasion and domination, and if it is a significant cause of loss to *S. salsa* habitat.
2. The rate and range of expansion patterns of *S. alterniflora* and *P. australis* in YNNR using a remote sensing time series analysis of 15 years (2003–2018).
3. Detect the critical year from deposition to erosion, and how much vegetation has been lost in the reserve area since erosion struck.

## 2 MATERIALS AND METHODS

### 2.1 Study area

YNNR is located on the east coast of Jiangsu Province, China, from 32°20′N to 34°37′N and 119°29′E to 121°16′E. The YNNR stretches out through five counties of Yancheng City, namely: Xiangshui County, Binhai County, Sheyang County, Dafeng City, and Dongtai County. For the significance and roles played by YNNR in biodiversity conservation, it has been recognized and enlisted in the world's prestigious wetlands list (Fang et al., 2012; Li et al., 2017). YNNR is sub‐divided into three zones: the core zone, buffer zone, and experimental zone. Figure 1 shows the location of YNNR core zone, and the three major saltmarshes found in the core zone are *Spartina alterniflora*, *S. salsa*, and *P. australis*. YNNR core zone has a total size of about 75 km^2^, whereas YNNR zones span out to about 570.33 km^2^ area of the landmass. The average annual precipitation in YNNR is 1,010 mm, and the annual average temperature is between 13.7 and 14.8°C. It is dry and cold in the winter, hot and rainy in the summer, probably because it lies in the transition belt between the northern subtropical zones and warm temperate.

**FIGURE 1:**
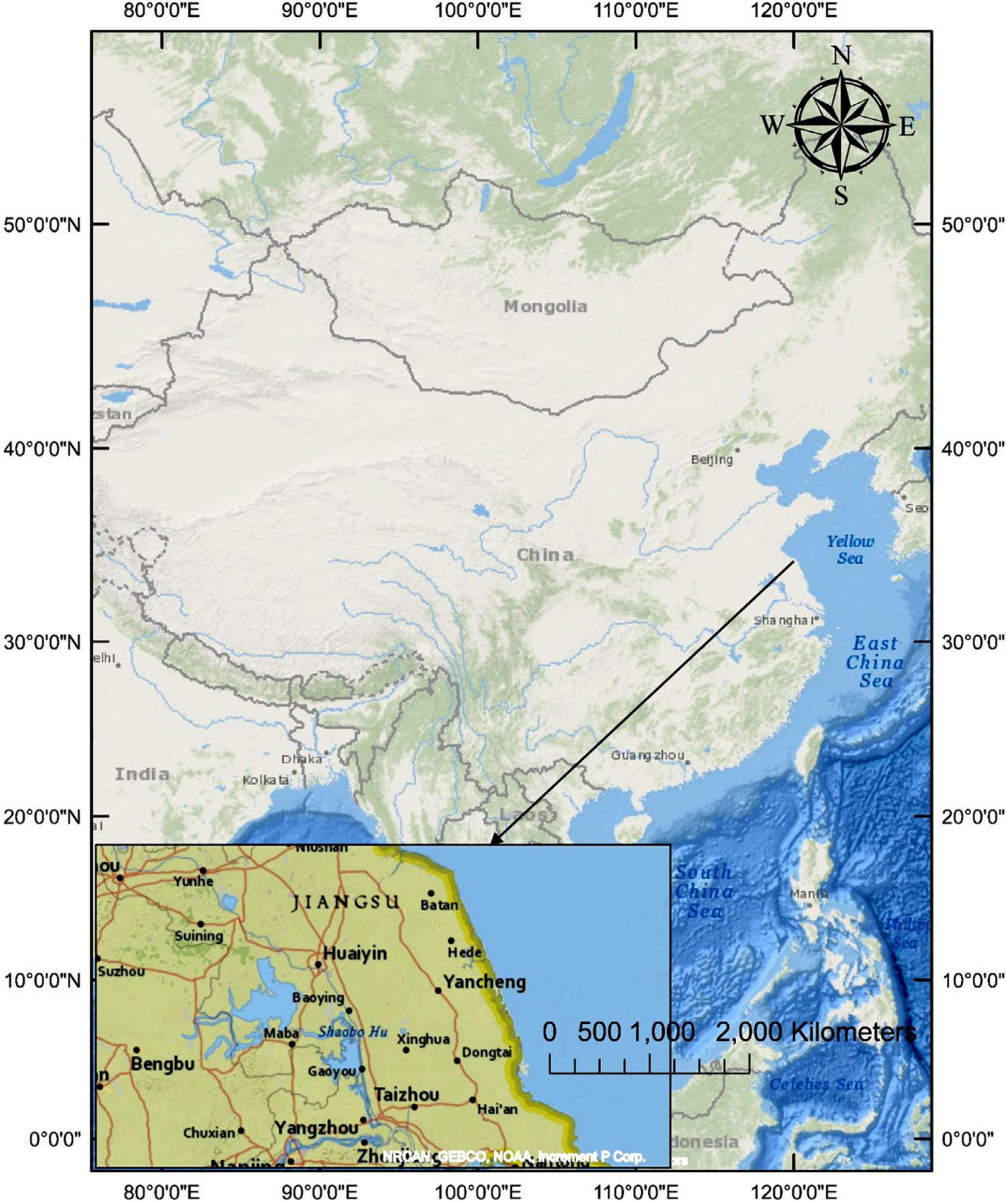
Map of China showing the location of our study area

### 2.2 Data acquisition

Figure 2 shows the workflow diagram used in solving our scientific problems in YNNR.

**FIGURE 2:**
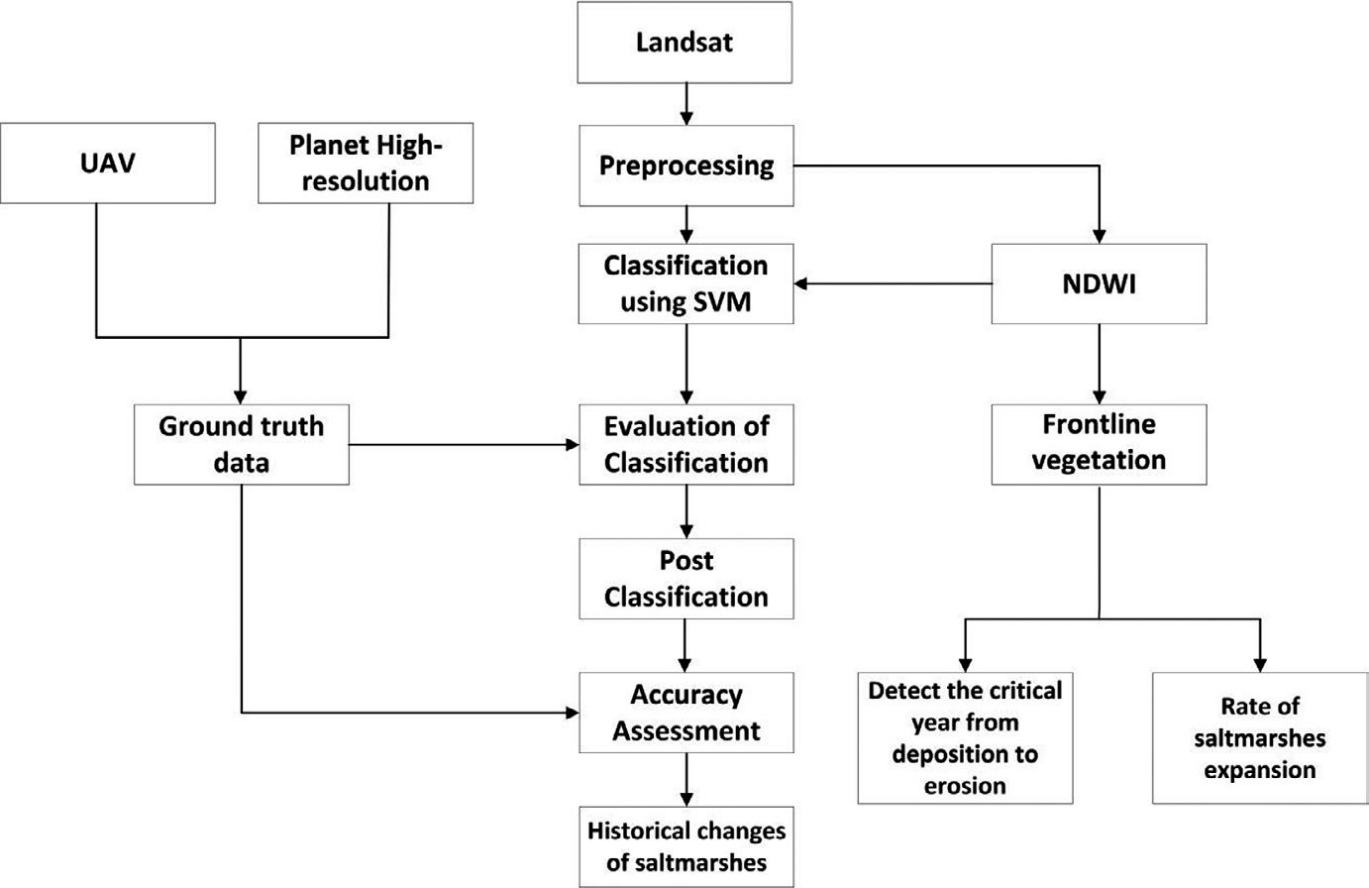
Work flow model

Ortho‐rectified Landsat TM (Thematic Mapper), ETM+ (Enhanced Thematic Mapper Plus), and OLI (Operational Land Imager) remotely sensed Landsat imagery were acquired from earthexplorer.usgs.gov for our studies. The data acquired were of low tides and similar seasons (February–March and July–September). Images of vegetation at a growing season and dormant season have different densities and useful in minimizing spectral confusion between *P. australis* and *S. alterniflora*. Landsat Imagery acquired was of the year 2003–2018, 15 (fifteen) scenes were carefully selected, and these show details of 15 years annual time series of the expansion and retraction of *S. alterniflora* and other native saltmarshes in YNNR. All cloud‐free and significant contrast between *S. alterniflora* and other salt marshes are in YNNR.

We also acquired high‐resolution satellite imagery from Planet's dove constellation www.planet.com. Planet's dove has ranges of nanosatellites that are deployable, providing a spatial resolution of 3–5 m and three spectral bands Red: 610–700 nm, Green: 500–590 nm, Blue: 420–530 nm, NIR: 770–900 nm (Planet scope, 2020). Planet's dove is meant to be low‐cost, rapidly deployable and can take pictures of the Earth on a 24‐hr basis.

### 2.3 Ground truth data acquisition and survey

YNNR core zone is heavily restricted and has a firm policy on trespassing, human interference or any human‐induced anthropogenic activities. Obtaining a ground truth data and access to the terrain was difficult. Furthermore, unmanned aerial vehicle (UAV) was flown in the zone, and we acquired a high‐resolution satellite data from planet earth constellation. The high‐resolution imagery was used as one of the ground truth data and, as well, was thoroughly compared with data of UAV as shown in our work flow diagram in Figure 2 and Landsat Satellite imagery, with the help of visual interpretation and expert knowledge of vegetation distribution in YNNR terrain.

### 2.4 Data processing

Correction and Processing of Landsat and Planet data used for this research consist of various steps due to atmospheric and radiance effect on satellite imagery, which often requires image preprocessing (Hoepffner & Zibordi, 2009; Mitasova, 2005). First, we processed every image individually using radiometric calibration function to reduce errors associated with reflectance caused by atmospheric elements (water vapor, atmospheric dust, etc.), which cause imprecisions in the satellite images (Prieto‐Amparan et al., 2018) and interpretability of data quality (Minarík et al., 2019; Beisl et al., 2010).

We subsetted all acquired satellite imagery to display only the core zone of YNNR. All images are of low tides because tidal flats are fully exposed for a short time on low tides. This makes it easier to use NDWI (Normalized Differential Water Index) method in extracting and excluding the study area from open water. The NDWI is a tool widely used to delineate open water and terrestrial environments features and mapping out tidal flats (Liu et al., 2012; McFeeters, 1996; Murray et al., 2012). Near‐infrared (NIR) and any visible band is suitable for performing NDWI delineating of waterline and tidal habitats (Zhao et al., 2008). We calculated NDWI for each pixel with the following formula:

Green‐NIR/Green+NIR=NDWI

where the green and NIR are the radiances of the green and near‐infrared wavelengths (Murray et al., 2012).

### 2.5 Remote sensing interpretation of tidal flats

We used front vegetation line technique to know how much vegetation expanded toward the open tidal flat, and how much was lost after erosion struck. These techniques involve setting 2003 and 2018 boundary line as a reference, then delineating all acquired Landsat vegetation frontline boundaries manually from the open tidal flats after performing NDWI. This method enhances the monitoring of salt marsh frontline expansion, retraction, and transition of deposition to erosion on YNNR.

Classification of saltmarshes was carefully carried out to study the expansion and retraction trend or competitions among different saltmarshes in the reserve. The *P. australis* class has a complex spectral signature with *S. alterniflora*, which is confusing as *P. australis* can grow in different environments with different soil moisture content and salinity (Burdick et al., 2001; Srivastava et al., 2013; Yuan et al., 2013). The significant factors affecting the spectral signatures are vegetation density and water content (Katja Klančnik, 2015; Ling et al., 2017). We used false composite colors of Band 5, 4, 3 and 4, 3, 2 for Landsat 8/OLI and Landsat TM/ETM imageries, respectively, before classification. Then, we used the support vector machine (SVM) for our classification. SVM is a supervised statistical learning algorithm developed by Vapnic in 1979 (Shoesmith, 1984) for pattern recognition and estimating multidimensional functions (Shoesmith, 1984). SVM is a base classifier that can handle, rebalance, and resample training data (Bj Orn Waske, 2009).

After post‐classification, the images were further subsetted and masked out to extract different vegetation trend like *S. salsa* habitat loses, patches, and eroded tidal flats. These entire tasks were computed and carried out in ENVI 5.3 and ArcGIS 10.7 software. ENVI and ArcGIS software are geospatial software used to view, edit, analyze, mapping out, and disseminating geographic data and extracting meaningful information from remotely sensed imagery to make better decisions (Maguire, 2008).

### 2.6 Accuracy assessment

Accuracy assessment determines the quality of information derived from a remotely sensed data and can reduce redundancy and assures assessment precision of a remotely sensed data (Huang et al., 2017). These assessments are either for a quick comparison of remote sensed data and map (qualitative assessment) or for the attempt to quantify and identify the error (quantitative assessment) in a remotely sensed classified data to see if it all corresponds to ground truth data or what is on the ground (Training Manual Developed by CEGIS, USFS and BFD, 2014‐15 & A.T.M.P., 2013). Accuracy assessment is one of the most crucial parts in image classification. For accuracy assessment to be sure, we used an error matrix method (confusion matrix) in performing accuracy assessment. Accuracy assessment is concerned with the correspondence between the class label and “true” class (Anupam, 2017; Anupam Anand, 2012). Here, our “true” class is from UAV and high‐resolution planet data. We obtained sufficient data for each vegetation in YNNR via the use of UAV and high‐resolution satellite. Furthermore, we used a random sampling method approach which is incorporated in our ENVI software after the geospatial analysis has been conducted, and we compare the results of our classified data to the reference data. Random sampling helps in generating a random sampling of points from a classification/classified result; random samplings are valuable in supporting classification accuracy assessments and ground truthing and in producing a representative sample by merely eliminating any possible voluntary response bias and guarding against any under coverage bias. Moreover, the accuracies such as producers’ accuracy, user's accuracy, and overall accuracy were calculated from the error matrix.

## 3 RESULTS

### 3.1 Frontline vegetation changes

The result from our acquired satellite data of 2003 shows the total area covered by all salt marshes in YNNR in 2003 was 64.372 km^2^. At present (2018), the total vegetation of all salt marshes land cover in YNNR is about 76.816 km^2^, showing an increase of about 12 km^2^ throughout the study period. After overlaying frontline vegetation of all satellite images, we found out that the frontline vegetation stops expanding toward the open tidal flats of YNNR from 2010 to 2018. However, the result also displays there was a rapid expansion of the saltmarshes frontline from 2003 to 2008. Hence, 2009 was the striking erosion year, as shown in Figure 3. This erosion currently appears on the vegetation front line of YNNR. After the erosion on the frontline vegetation struck, about 5.0 km^2^ of salt marshes has been lost to erosion.

**FIGURE 3:**
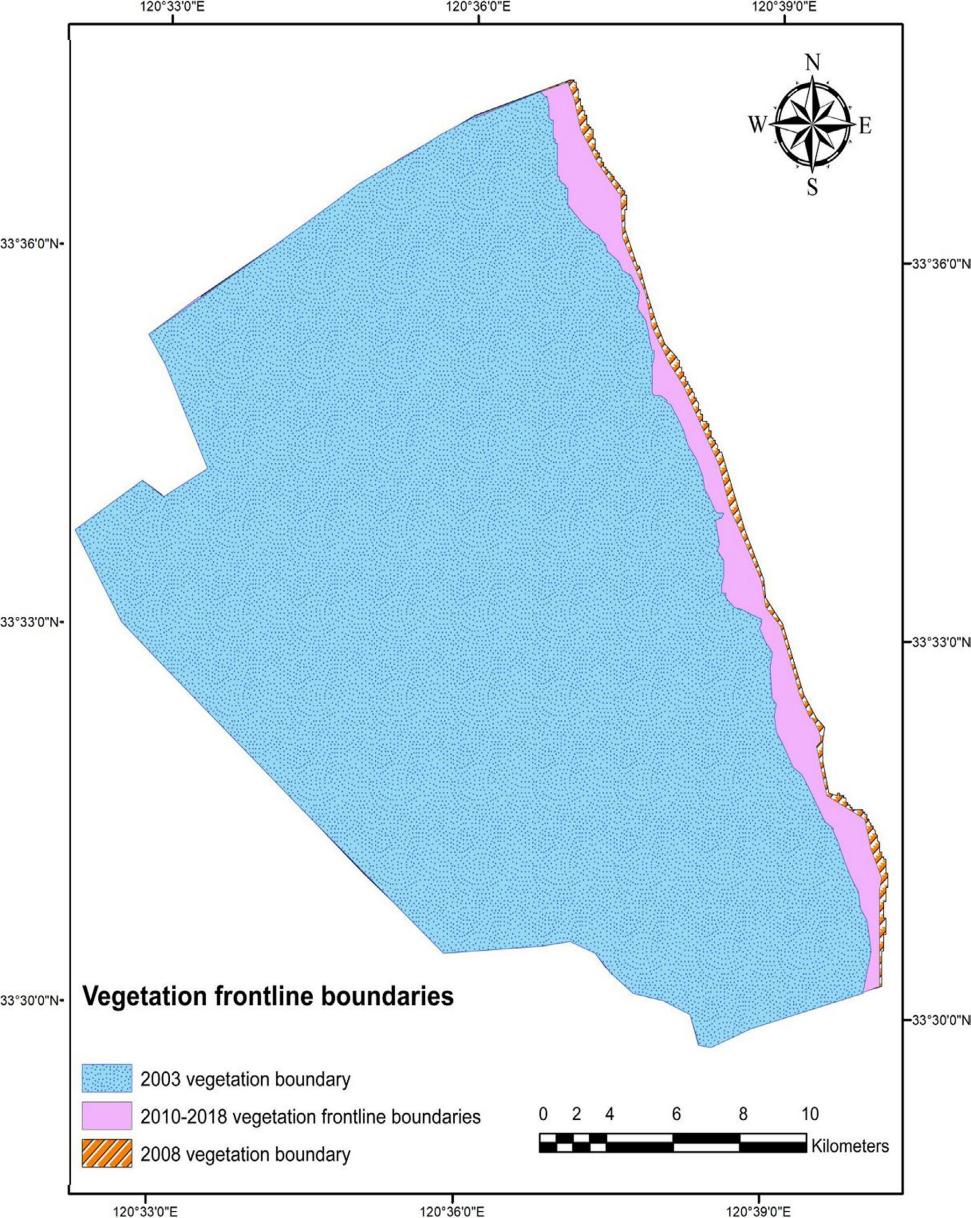
Historical shoreline vegetation boundaries changes in YNNR core zone

### 3.2 Classification of salt marshes

The overall accuracy of our classification shows that the intertidal salt marsh maps accuracy stood at 95.53%, as shown in Table 1. The red‐crowned crane habitat *S. salsa* has most of its habitat appeared to be in patches and segments after classifications. Both *S. alterniflora* and native saltmarsh *P. australis* have contributed equally in the habitat loss of *S. salsa*, as shown in Figure 4.

**TABLE 1:** Accuracy assessment of SVM

Class	Others	*Spartina alterniflora*	*Suaeda salsa*	*Phragmites australis*	Total
Ground truth (Pixels)					
Unclassified	0	0	0	0	0
Others	28	4	0	1	33
*Spartina alterniflora*	0	26	0	0	26
*Suaeda salsa*	0	0	24	0	24
*Phragmites australis*	0	0	0	29	29
Total	28	30	24	30	112
Ground truth (Percent)					
Unclassified	0.00	0.00	0.00	0.00	0.00
Region #1	100.00	13.33	0.00	3.33	29.46
Region #2	0.00	86.67	0.00	0.00	23.21
Region #3	0.00	0.00	100.00	0.00	21.43
Region #4	0.00	0.00	0.00	96.67	25.89
Total	100.00	100.00	100.00	100.00	100.00

Class	Commission (Percent)	Omission (Percent)	Commission (Pixels)	Omission (Pixels)
Others	15.15	0.00	5/33	0/28
*Spartina alterniflora*	0.00	13.33	0/26	4/30
*Suaeda salsa*	0.00	0.00	0/24	0/24
*Phragmites australis*	0.00	3.33	0/29	1/30

Class	Producer's accuracy (Percent)	User's accuracy (Percent)	Producer's accuracy (Pixels)	User's accuracy (Pixels)
Others	100.00	84.85	28/28	28/33
*Spartina alterniflora*	86.67	100.00	26/30	26/26
*Suaeda salsa*	100.00	100.00	24/24	24/24
*Phragmites australis*	96.67	100.00	29/30	29/29

Note

Kappa Coefficient = 0.9404. SVM Overall Accuracy = (107/112) 95.5357%.

**FIGURE 4:**
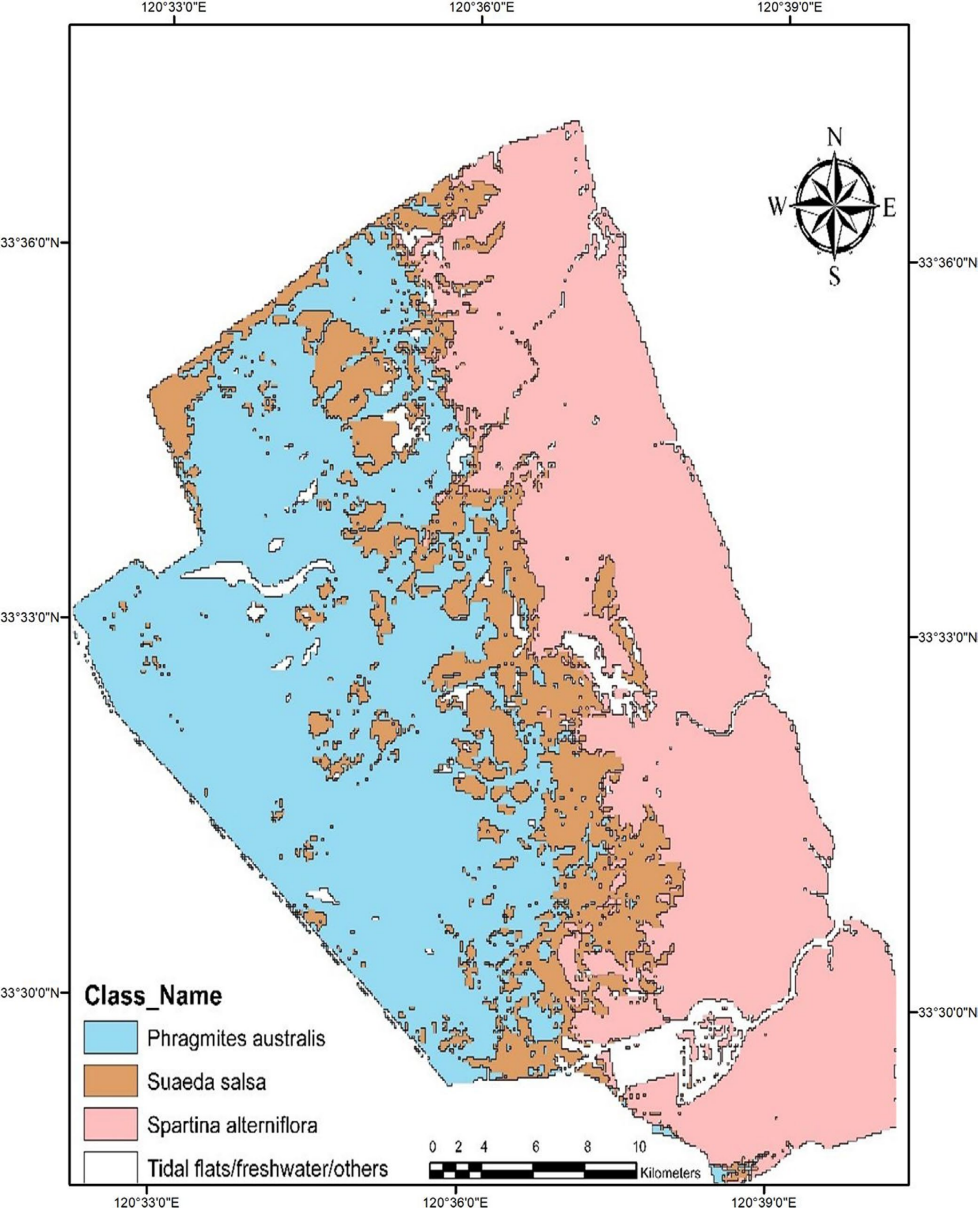
The current vegetation status in YNNR

### 3.3 Saltmarshes historical competition and changes in YNNR

The growth of *S. alterniflora*, as recorded in 2003, was spreading toward the sea, as shown in Figure 5. The growth and spread of *S. alterniflora* were consistent and uniform until the year 2009. Our classified satellite data show a shift in the biome and backwards or retraction growth toward *S. salsa* and the encroachment of *P. australis* from the inland of YNNR core zone toward *S. salsa*. However, *S. salsa* habitat in YNNR is prone to shrinking and increased landscape fragmentation due to the invasion by *S. alterniflora* and its local saltmarsh counterpart *P. australis*. This landward shift led to the further reduction and fragmentation of *S. salsa* and its fragile habitat.

**FIGURE 5:**
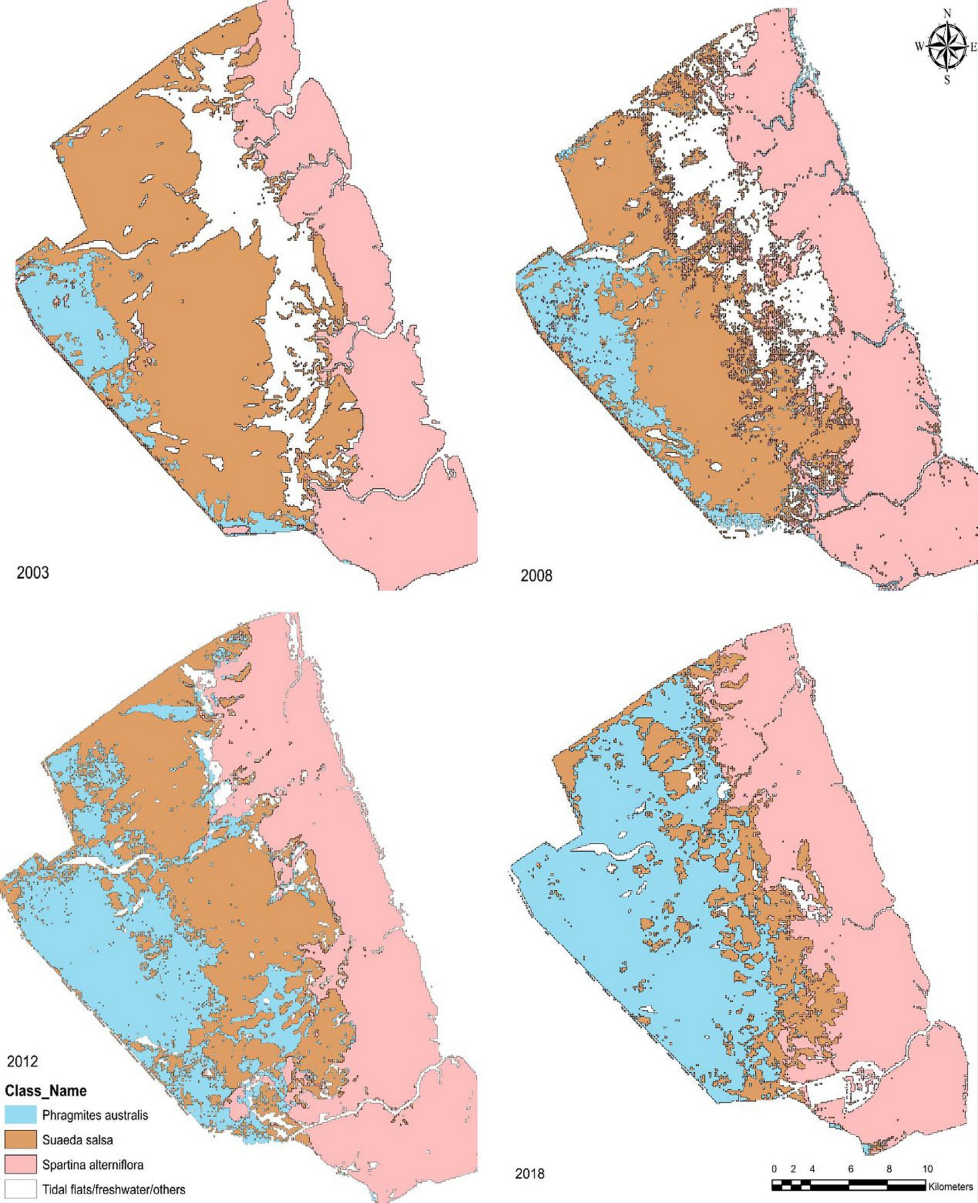
YNNR vegetation habitat changes

In 2003, *S. alterniflora* in YNNR core zone covered a total area of 22.067 km^2^, while *S. salsa* the most preferred habitat for migratory waterbirds was the chief dominating vegetation and covered an area of 37.553 km^2^. Thus, *P. australis*, a native saltmarsh in the zone, covers a small portion of 4.752 km^2^. Currently (2018), *S. salsa* shrank and reduced drastically to a limited space of 10 km^2^, which is quite alarming. *Spartina alterniflora* grew from 22.067 km^2^ in 2003 to 31.113 km^2^ in 2009, thus exhibiting the core characteristics of the invasive grass, “invading and dominating.” Whereby *P. australis* currently occupied a total area of 32.792 km^2^ from 4.752 km^2^ in 2003 in YNNR core zone, this shows *P. australis* expansion is as substantial as *S. alterniflora* and also a significant dominator in YNNR core zone as shown in Figure 6. *Phragmites australis* has a dense growth, thereby, supplanting *S. salsa* from the inland zone and moving toward *S. alterniflora* dominated area.

**FIGURE 6:**
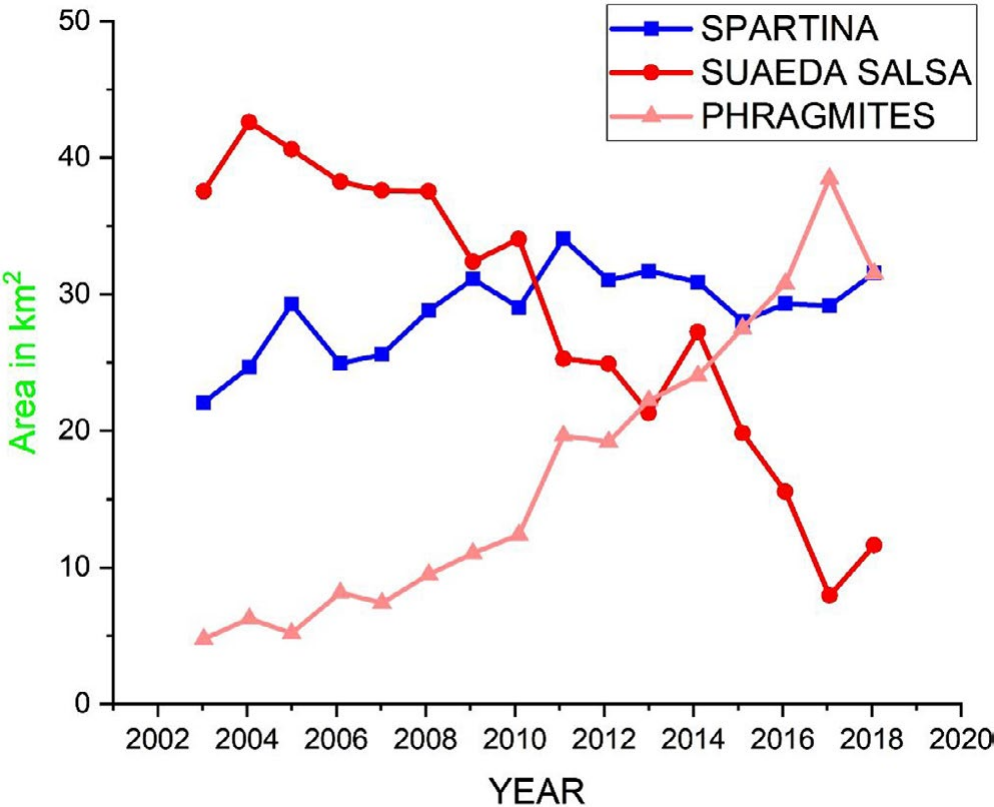
Domination and stability graph of *Spartina alterniflora*, *Phragmite australis* to *Suaeda salsa* in YNNR core zone

Also, *S. salsa* lost 25.99 km^2^ of its entire land cover in YNNR to *S. alterniflora* and *P. australis* throughout the whole study period (2003–2019). The current rate of loss and the high incidence of degrading processes suggest that *S. salsa* habitat is at risk of being lost and might lead to extinction in YNNR core zone. The average annual increase in the width of *S. alterniflora* was 86.39 m per annum. *S. alterniflora* growth was recorded and observed to be stable, average, and a bit stagnant (starting from 2009, when the shoreline erosions were detected), thus gradually encroaching the habitat of *S. salsa*.

### 3.4 Changes in the growth pattern of *S. alterniflora* and its effect on red‐crowned crane habitat

In 2009, we observed a sudden change and retraction pattern of *S. alterniflora*. Our remote sensed data result displays some retraction and backward or landward growth of *S. alterniflora* toward *S. salsa* onshore of YNNR. This landward shift led to the severe reduction of *S. salsa* fragile habitat as it appeared to be in severe patches (Figure 7).

**FIGURE 7:**
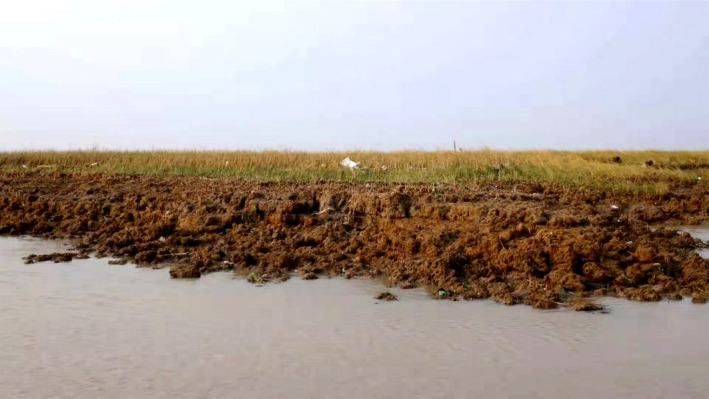
Current cliff erosion in YNNR Core zone

## 4 DISCUSSION

*Spartina alterniflora*, just like any other invasive species, is an additional burden and threat, which has led to biotic and abiotic degradation of tidal flats ecosystem (Fuller & Muller, 2015). The sequence of biodiversity loss in YNNR can be pointed to (1) movement or introduction of invasive species, (2) distribution and disturbance of ecosystems, (3) biotic exchange, changes, and loss of habitat, (4) increased competition and extinction, and (5) biodiversity loss.

The invasion of *S. alterniflora* in South and Eastern China is said to be the world's most massive invasion of such cordgrass species (Strong & Ayres, 2013). Our research shows that *S. alterniflora* has substantially reduced macrobenthic diversity in Jiangsu Province (Zhou et al., 2009) and has altered community structure for migratory birds and endangered species communities in YNNR.

*Spartina alterniflora* and *Phragmites australis* is currently an aggressive competitor in YNNR, outcompeting native floras and saltmarshes. In the early days, before the sporadic domination and invasion of *S. alterniflora*, when there is inundation, tides deposit a large number of nutrients in the *S. salsa* marsh habitat, providing a daily food source for benthic organisms. Thus, when *S. alterniflora* began to dominate and invade the coastal tidal flats of YNNR, *S. alterniflora* blocked the frequency of tidal immersion in *S. salsa* marshes—thereby limiting the flow of nutrients carried by the tide to continue to supply to *S. salsa* marsh. However, these actions result in the decrease of benthic biomass and species in *S. salsa* marsh and habitat. Thus, it also affected the food supply of cranes and increased the difficulty of foraging to a certain extent (Yang et al., 2015; Wei & Wenbing, 2020; Li & Ge, 2013), thereby giving room for enough freshwater to be retained in YNNR, which gives *P. australis* leverage in its expansion and domination of 32.792 km^2^ of the whole study area from the supratidal area.

The rapid vegetation cover changes of *S. alterniflora* and its retraction are characterized by several factors, including marsh erosion that occurred at the vegetation front line of *S. alterniflora* in YNNR shoreline, where *S. alterniflora* are dominant as reported by this research in YNNR. Researchers found out that one of the most contributing factors to the deterioration of saltmarshes and erosion are storms, wind and wave actions, wave height, beach elevation, cliff height, and total amount of wave power striking the shoreline (Schwimmer, 2001), hence the erosion in YNNR shoreline. Further studies into soil erosion and its prediction have indicated that soil texture parameters, soil structure, and permeability are linked to soil and saltmarshes erosion (Wang et al., 2016). Irrespective of the complexity surrounding erosion and eroded saltmarshes, salt marsh erosion continuously occurs, even under low and high wave energy conditions (Currin et al., 2018; Sharma et al., 2016; Li, 2019), and these wave's effects are mostly concentrated in the shoreline of YNNR. The consistency of this wave on the shoreline of YNNR will continue to erode the shoreline. Thus, the effect will cause *Spartina alterniflora* to grow inwards and squeeze *S. salsa* saltmarsh habitat from the intertidal zone, and *P. australis* will do the same from the supratidal zone.

### 4.1 Importance of wintering sites to Red‐crowned crane birds and their behavior in each of the Habitat

Wintering sites are of extreme importance for migratory birds like red‐crowned cranes (*Grus japonensis*) and also play a vital role during this season and their breeding period. Red‐crowned cranes are choosy, and their habitat preference is related to many criteria's such as availability of food (Alonso et al., 1994), disturbance agents (Cao & Liu, 2008; Wang et al., 2011), sleeping, and resting places. *Suaeda salsa* vegetation is essential and vital in the nesting, roosting, and foraging habitat for the red‐crowned crane in YNNR (Li et al., 2014). Reasons are the abundance of nutrient‐rich tidal mud‐flat crabs, fish, insects, aquatic invertebrates, amphibians, rodents, reeds, heath berries grasses, corn, and other plants that occupy the habitat as red‐crowned crane's diet consists of all the predominant food resources listed above (Li et al., 2014; Red‐crowned‐crane, 2018). *Suaeda salsa* vegetation in YNNR has the highest red‐crowned crane presence of all the land cover types in YNNR (Peng et al., 2020; Xu, 2013).

However, red‐crowned cranes source their proteins along the rivers and ponds as it provides an abundance of it. Also, these rivers and ponds provide drinking water and resting sites for them; these sites are seen in *S. salsa* vegetated area and where *P. australis* and *S. salsa* share boundaries or mixed up. Red‐crowned cranes usually and always avoid dense and high forests and salt marshes (Li et al., 2017; Masatomi & Masatomi, 2018) like *S. alterniflora* and tall *P. australis* as observed in YNNR. These sites can be a habitat or hiding place for predators and invaders, also can be an obstacle to them during flights and landing. Red‐crowned cranes prefer areas with ample space and visibility where they can easily see, sense, and detect predators like the wildcats (*Felis bengalensis euptilura*) (Kim et al., 2016). *Suaeda salsa* vegetation provides a good view of the terrain, without igniting the fear to engage in frequent head‐up vigilance, but has shrunk to a little space of 10 km^2^. Red‐crowned cranes need to continually have to maintain and increase their intake capacity to meet their energy budget (Liu et al., 2020; Li & Ge, 2013). This is important because of their minimal temporal window of foraging opportunities.

Habitat loss and habitat shrinkage are considered the greatest threat to biodiversity (Wilson et al., 2016). The impact in the change of habitat configuration and habitat fragmentation has an overall negative effect on the genetic diversity of organisms and decreases population size (Fletcher et al., 2018). Fragmentation of a habitat occurs when large habitat is transformed into smaller patches of smaller total area from a matrix of habitat unlike the origin (Baur, 2018). Habitat loss can disrupt Individuals of an isolated population in interactions among species (Soga & Koike, 2012). It could also change or disrupt antagonistic and mutualistic interactions, such as predation or parasitism (Bordes et al., 2015). With their interspecific competition and pollination (Kolb, 2008; Sozio & Mortelliti, 2016), many animals and bird species are quite sensitive to their habitat and when isolated, and may lose species at a high rate (Fletcher et al., 2018), thus the low influx of avian populations in YNNR.

### 4.2 Current state of YNNR coastal tidal flat and transferrable context of our method to wetland functions and loss

Yellow sea tidal flats have been subjected to sustained coastal squeeze for several decades. This phenomenon has lingered and is driven by extensive coastal development, sea‐level rise, coastal subsidence, compaction, and erosion sediments that flow from the two major rivers in China, the Yellow River (Huang He), and the Yangtze River (Chang Jiang) flowing into the yellow sea has declined by more than 90% and 70%, respectively, over the last ten decades. Sediment discharge from these rivers is critical and essential for the reformation and refurbishment of tidal flats following seasonal erosion. It enables tidal flats to be resilience with the rising sea levels has a record of critical declination over the past five decades (Syvistki et al., 2005; Wu & Jiangguo, 2015).

This study is aimed at providing a quantitative analysis in the impact of acquiring multispectral and high‐resolution remote sensed imagery in the study and monitoring of habitat loss, habitat shrinkage, and its dynamics and in detecting any changes in any land cover and its causes, especially in a dedicate environment such as YNNR. The methods used in this paper can be easily transferred to the assessment, tracking and monitoring habitats changes and loss using remote sensing at large (regional) scales, the effects of invasive species (at large (regional) scales) on wetland bird habitat and wetland function (at large (regional) scales). However, high‐resolution imagery and support vector machine classification can be able to clarify the confusion of various multispectral complex spectral signatures of vegetation. They can as well show clearly the grow back, vegetation extent growth and loss, with the deterioration of vegetation along the shoreline in complex and restricted places like YNNR, also necessitating comparisons with historical data sand topographic maps in order to have a suitable long‐term assessment of ecosystem change (Murray, Allen, et al., 2014; Murray, Clemens, et al., 2014). These call for further research on the study of wetland restoration and management, wave energy, currents, tides, winds, sand sources, and sinks before its effect in YNNR and how to mitigate the menace and actions in YNNR. This study predicts the fact that *S. alterniflora* will continue to retract and grow inwards the YNNR, while *P. australis* will encroach the habitat from the sup thereby displacing the wintering habitat quality of migratory red‐crowned cranes, their breeding and foraging habitat in YNNR core zone.

## CONFLICTS OF INTEREST

None declared.

## AUTHOR CONTRIBUTION

**Onyedikachi Kingsley Okoye:** Data curation (equal); Investigation (equal). **Huan Li:** Project administration (equal); Resources (equal); Supervision (equal). **Zheng Gong:** Funding acquisition (equal); Project administration (equal); Supervision (equal).

## Data Availability

Data are available on: earthexplorer.usgs.gov (Gov EU). I agree to deposit my data to public repository. Sampling locations‐Yancheng National Nature Reserve. Dryad: http://doi.org/10.5061/dryad.1g1jwstrm.
